# Modulation of Tim-3 Expression by Antigen-Dependent and -Independent Factors on T Cells from Patients with Chronic Hepatitis B Virus Infection

**DOI:** 10.3389/fcimb.2017.00098

**Published:** 2017-03-28

**Authors:** Jie Dong, Xiao-Fei Yang, Lin-Xu Wang, Xin Wei, An-Hui Wang, Chun-Qiu Hao, Huan-Jun Shen, Chang-Xing Huang, Ye Zhang, Jian-Qi Lian

**Affiliations:** ^1^Center for Infectious Diseases, Tangdu Hospital, Fourth Military Medical UniversityXi'an, China; ^2^Department of Ophthalmology and Otorhinolaryngology, Tenth Hospital of PLAWuwei, China; ^3^Department of Epidemiology, School of Public Health, Fourth Military Medical UniversityXi'an, China

**Keywords:** hepatitis B virus, T-cell immunoglobulin domain and mucin domain-containing molecule-3, T cell receptor, common γ-chain cytokines, immunomodulation

## Abstract

T-cell immunoglobulin domain and mucin domain-containing molecule-3 (Tim-3) was up-regulated on viral specific T cells and contributed to T cells exhaustion during chronic hepatitis B virus (HBV) infection. However, modulation of Tim-3 expression was still not fully elucidated. To evaluate the potential viral and inflammatory factors involved in the inductor of Tim-3 expression on T cells, 76 patients with chronic HBV infection (including 40 chronic hepatitis B [CHB] and 36 asymptomatic HBV carriers [AsC]) and 40 of normal controls (NCs) were enrolled in this study. Tim-3 expressions on CD4^+^ and CD8^+^ T cells were assessed in response to HBV-encoding antigens, HBV peptide pools, and common γ-chain (γc) cytokines stimulation by flow cytometry. HBV peptides and anti-CD3/CD28 directly induced Tim-3 expression on T cells. γc cytokines also drive Tim-3 up-regulations on both CD4^+^ and CD8^+^ T cells in patients with chronic HBV infection. However, γc cytokines did not enhance the Tim-3 inductions by either anti-CD3/CD28 or HBV peptides stimulation. Furthermore, γc cytokines-mediated Tim-3 induction could not be abrogated by γc cytokine receptor-neutralizing antibodies. The current results suggested that elevation of Tim-3 expression on T cells could be regulated by both antigen-dependent and -independent manner in patients with chronic HBV infection. The role of γc cytokines in modulation of inhibitory pathway might be evaluated as immunotherapies in humans.

## Introduction

Hepatitis B virus (HBV) leads to a chronic infection in 10% of adults and 90% of children, which results in 1~2 million people died annually worldwide due to HBV-related end-stage liver diseases, such as liver cirrhosis, hepatic failure, and hepatocellular carcinoma (Hoofnagle et al., 2007; Lok and McMahon, 2009; Lu and Zhuang, 2009). The outcome of hepatitis B is closely linked to their immune status to mediate the clearance of virus. Interferon-γ (IFN-γ) production by viral specific CD4^+^ and CD8^+^ T cells response is pivotal for controlling acute hepatitis B virus infection (Rehermann et al., 1995; Bertoletti and Naoumov, 2003). In contrast, the inability of T cells results in the collapse of HBV-specific adaptive immune response in chronic hepatitis B (CHB) (Bertoletti and Naoumov, 2003; Chisari et al., 2010). More importantly, chronic HBV infection is not directly associated with liver inflammation, which is the results of interaction between virus and host immune response. Chronic HBV infection could be divided into different phases (Lian et al., 2014). Immune tolerant phase is characterized by high HBV DNA and normal ALT, which showed as asymmetric HBV carriers, while CHB patients reveal acute increase in ALT and continuing hepatic injury (Lian et al., 2014). However, the precise mechanisms corresponding to T cells tolerance and immune evasion in chronic HBV infection are still not fully elucidated.

Recent studies revealed that multiple inhibitory immune regulatory proteins, including programmed death-1 (PD-1), cytotoxic T-lymphocyte antigen-4 (CTLA-4), and T-cell immunoglobulin domain and mucin domain-containing molecule-3 (Tim-3), were involved in the modulation of T cells impairment during chronic infections (Seddiki et al., 2014; Pauken and Wherry, 2015a,b). Tim-3 could be expressed on several cell types in immune system, including CD4^+^ and CD8^+^ T cells (Monney et al., 2002; Hastings et al., 2009; Dorfman et al., 2010). The role of Tim-3 could be vary depending on contexts where it was expressed (Gorman and Colgan, 2014). Study on tuberculosis infection provided evidence that Tim-3 promoted both CD4^+^ and CD8^+^ T cell responses (Qiu et al., 2012). However, Tim-3 was found to strongly suppress the T cells functions and was associated with T cells impairment or exhaustion in autoimmune diseases (Lee and Goverman, 2013) and chronic microbial infections (Jones et al., 2008; Sehrawat et al., 2009; Moorman et al., 2012; Gorman et al., 2014). Furthermore, Tim-3 contributed to T cell exhaustion partly by enhancing T cell receptor (TCR)-signaling pathway (Wherry, 2011; Ferris et al., 2014), while TCR was also an essential component of Tim-3 elevation based on the finding that CD3/CD28 costimulation up-regulated Tim-3 expression on CD4^+^ T cells (Hastings et al., 2009).

Overexpression of Tim-3 contributed to HBV persistence by induction of T cells dysfunction (Li et al., 2012; Nebbia et al., 2012), inhibition of viral-specific CD8^+^ T cells (Ju et al., 2009; Wu et al., 2012), and suppression of natural killer cells (Ju et al., 2010). However, the role of elevated Tim-3 on T cells in chronic HBV infection was still poorly understood. Human immunodeficiency virus (HIV)-1 protein Nef directly induced PD-1 expression, which was another exhaustion marker (Muthumani et al., 2008). Moreover, common γ-chain (γc) cytokines also induced Tim-3 expression in an antigen-independent manner in HIV-1 infection (Mujib et al., 2012). Thus, we hypothesized that soluble viral and inflammatory factors may be involved in the inductor of Tim-3 expression on T cells. To test this possibility, Tim-3 expressions on CD4^+^ and CD8^+^ T cells were examined in response to HBV antigens, peptides, or γc cytokines stimulation. The synergic effects of these factors were also evaluated by costimulation.

## Methods

### Subjects

A total of 76 hepatitis B e antigen (HBeAg)-positive HBV-infected patients, including 40 CHB patients and 36 asymptomatic HBV carriers (AsC), were enrolled in this study. The diagnoses were made according to the diagnostic standard of Chinese Guideline of Prevention and Treatment for Chronic Hepatitis B (2010 version). All patients were hospitalized or followed-up in Tangdu Hospital from March 2011 to July 2014. No patients were co-infected with HIV, other hepatitis viruses, or concurrently afflicted by autoimmune diseases. Patients who previously received anti-HBV agents or immunomodulatory treatments were also excluded. For normal controls (NCs), Forty healthy individuals with matched age and sex were also enrolled. The clinical data obtained for the enrolled subjects are listed in Table 1. The study protocol was approved by the ethics committee of Tangdu Hospital, Fourth Military Medical University, and written informed consent was obtained from each subject.

**Table 1:** **Clinical characteristics of enrolled subjects**.

	**NC**	**AsC**	**CHB**
Case (*n*)	40	36	40
Age (years)	24.52 ± 4.02	25.52 ± 3.09	26.15 ± 2.18
Male gender (%)	62.50% (25/40)	52.78% (19/36)	67.50% (27/40)
HBV DNA (log10 IU/mL)	N.A.	4.53 ± 1.70	4.01 ± 1.95
ALT (U/L)	15.26 ± 3.11	23.54 ± 5.51	87.00 ± 24.35

*NC, normal controls; AsC, asymptomatic HBV carrier; CHB, chronic hepatitis B; N.A., not applicable. Data were presented as mean ± SD*.

### Virological and biochemical assessments

HBV DNA was quantified using a commercial real-time PCR kit (PG Biotech, Shenzhen, China) with a detection limitation of 2 log10 copies/mL. Hepatitis B surface antigen (HBsAg), anti-HBs, HBeAg, anti-HBe, anti-hepatitis core antigen were quantified using the ARCHITECT HBsAg, anti-HBs, HBeAg, anti-HBe, and anti-HBc reagent kit (Abbott GmbH & Co. KG, Wiesbaden, Germany). Serum biochemical assessments were made using an automatic analyzer (Hitachi 7170A, Hitachi Ltd, Tokyo, Japan).

### Peripheral blood mononuclear cells (PBMCs) isolation and stimulation

PBMCs were isolated by Ficoll-Hypaque (Sigma-Aldrich, St. Louis, MO) density gradient centrifugation. PBMCs were cultured at 1 × 10^6^/mL in RPMI 1640 (Invitrogen Gibco, Grand Island, NY, USA) supplemented with 10% heat-inactivated fetal bovine serum (FBS; Invitrogen Gibco). PBMCs from CHB and AsC were stimulated with either the mixture of HBV antigens [including HBsAg (AbDSerotec, Oxford, UK; final concentration 5 μg/mL), HBeAg (Abcam, Cambridge, MA, USA; final concentration10 μg/mL), and HBcAg (AbDSerotec; final concentration1 μg/mL)] or the mixture of HBV full-genome peptides pool (15 amino acids of each peptide with 5 amino acids overlapping, final concentration 10 μg/mL) for 4 days. In some experiments, PBMCs were also co-cultured with either common γc receptor cytokines (IL-2, IL-7, IL-15, or IL-21, respectively, Peprotech Inc, Rocky Hill, NJ, USA; final concentration 25 ng/mL), anti-CD3/CD28 (eBiosciences, San Diego, CA, USA; final concentration 1 μg/mL), or anti-common γc receptor neutralizing antibody (R&D System, Minneapolis, MN, USA; final concentration 10 μg/mL) for 4 days. The concentrations of the antigens, peptides, cytokines, and neutralizing antibody were reported previously (Zhang et al., 2010, 2014; Mujib et al., 2012; Zhao et al., 2015; Wei et al., 2016).

### Flow cytometry

Anti-CD3-APC (BD Bioscience, San Jose, CA, USA), anti-CD4-FITC (BD Bioscience), anti-CD8-PE-Cy7 (BD Bioscience), and anti-Tim-3-PerCP-Cy5.5 (R&D System) were used for surface staining. In some experiments, anti-phosphorylated STAT1 (p-STAT1)-Alex Flour 647 (pY701) (BD Bioscience) was also used for intracellular staining. Samples were analyzed with a BD FACS Aria II analyzer (BD Biosciences). Acquisitions were performed with CellQuest Pro software (BD Biosciences) and analyses were performed with FlowJo version 7.6.2 for Windows (Tree Star Inc., Ashland, OR, USA). Isotype control antibodies were used to separate positive and negative cells in the Alex Flour 647, APC, FITC, PE-Cy7, and PerCP-Cy5.5 fluorescence channels.

### Statistical analysis

Data were analyzed using Graphpad Prism version 5.0 (GraphPad Software, La Jolla, CA, USA). The Kruskal-Wallis H test and Dunn's multiple comparison test were used for comparison among groups. The Mann-Whitney test was used for comparison between two groups. A value of *P* < 0.05 was considered to indicate a significant difference.

## Results

### HBV peptides directly induced Tim-3 expression on T cells

**Figure 1:**
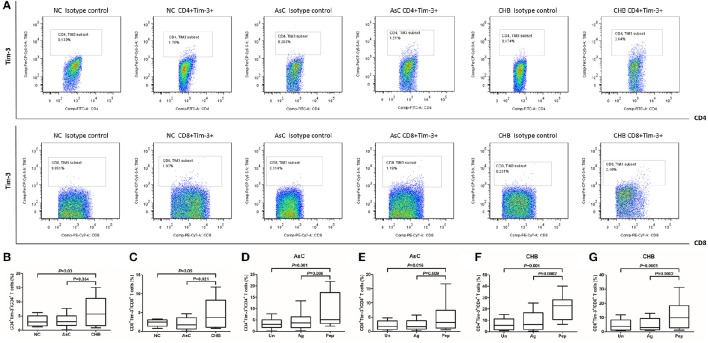
**Tim-3 expression on CD4^+^ and CD8^+^ T cells in response to HBV-encoding antigens and HBV peptide pools**. PBMCs from all enrolled subjects (including 40 of NCs, 36 of AsC, and 40 of CHB) were stained and tested. **(A)** Representative flow plots of Tim-3^+^ cells within CD4^+^ and CD8^+^ T cells in normal control (NC), asymptomatic HBV carrier (AsC), and chronic hepatitis B (CHB). PerCP-Cy5.5 Isotype control was used in each analysis for the separation of Tim-3-positive and -negative population. Comparison of frequencies for CD4^+^Tim-3^+^ **(B)** and CD8^+^Tim-3^+^ cells **(C)** in NCs, AsC, and CHB. Thus, the frequencies of Tim-3 expression on unstimulated T cells were used as controls for further studies. Comparison of frequencies for CD4^+^Tim-3^+^ **(D)** and CD8^+^Tim-3^+^ cells **(E)** in response to HBV-encoding antigens and HBV peptide pools stimulations for 4 days in AsC. Comparison of frequencies for CD4^+^Tim-3^+^ **(F)** and CD8^+^Tim-3^+^ cells **(G)** in response to HBV-encoding antigens and HBV peptide pools stimulations for 4 days in CHB. Data were presented as box-and-whisker plot. The box presented as median and quartile, and the whisker plot presented as 2.5–97.5% percentile. Dunn's multiple comparison test were used for comparison between groups.

We firstly analyzed the difference of Tim-3 expression on T cells between NCs and HBV-infected individuals. PBMCs from all enrolled subjects (including 40 of NCs, 36 of AsC, and 40 of CHB) were stained and tested. Representative PBMC samples from NC, AsC and CHB analyzed by flow cytometry was shown in Figure 1A. PerCP-Cy5.5 Isotype control was used in each analysis for the separation of Tim-3-positive and -negative population. Elevated expression of Tim-3 on both CD4^+^ (6.41 ± 5.00%) and CD8^+^ (4.72 ± 3.98%) T cells was found in patients with CHB in comparison with AsC (CD4^+^, 3.35 ± 2.22%, *P* = 0.034, Figure 1B; CD8^+^, 2.06 ± 1.63%, *P* = 0.021, Figure 1C) and NC (CD4^+^, 3.32 ± 1.83%, *P* = 0.03, Figure 1B; CD8^+^, 2.28 ± 0.94%, *P* = 0.049, Figure 1C).

Previous study demonstrated that HIV-1 viral products could not directly induced Tim-3 expression on T cells (Mujib et al., 2012). However, it was possible that the activity of HBV viral products might differ due to strong immunogenicity of HBV antigens. Thus, we then analyzed the Tim-3 expression on CD4^+^ and CD8^+^ T cells in response to either HBV antigens or peptides pool. Frequencies of Tim-3 expression on unstimulated T cells from AsC and CHB, which were presented in Figures 1B,C, were used as controls for further analysis. Mixture of HBsAg, HBeAg, and HBcAg did not up-regulate the expression of Tim-3 on T cells in either AsC (*P* > 0.05, Figures 1D,E) and CHB (*P* > 0.05, Figures 1F,G). Interestingly, HBV peptides pool could strongly induce increased expression of Tim-3 on both CD4^+^ and CD8^+^ T cells, with approximately elevation of 2.5-fold in AsC (CD4^+^, 8.77 ± 7.41%, *P* = 0.001, Figure 1D; CD8^+^, 5.44 ± 5.50%, *P* = 0.016; Figure 1E) and 3.5-fold in CHB (CD4^+^, 21.33 ± 10.25%, *P* = 0.001, Figure 1F; CD8^+^, 11.16 ± 9.04%, *P* = 0.0001; Figure 1G). There were no remarkable correlation between Tim-3 expression on T cells and HBV DNA or ALT levels in CHB and AsC (*P* > 0.05).

### The common γc cytokines drive Tim-3 expression on CD4^+^ and CD8^+^ T cells in patients with chronic HBV infection

**Figure 2:**
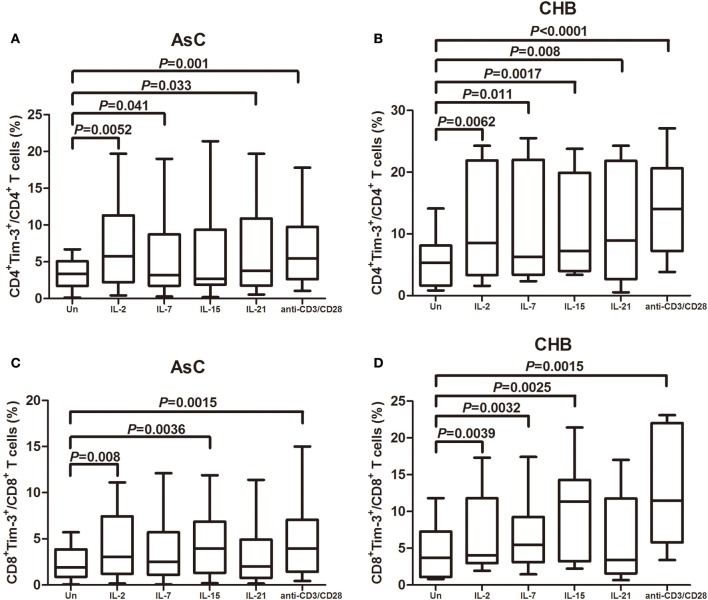
**Common γ-chain (γc) cytokine-mediated induction of Tim-3 expression in CD4^+^ and CD8^+^ T cells contained in peripheral blood mononuclear cells (PBMCs)**. PBMCs were selected from 18 of AsC and 20 of CHB, which were used in the experiments of Figure 1. Total PBMCs were treated with IL-2 (25 ng/mL), IL-7 (25 ng/mL), IL-15 (25 ng/mL), IL-21 (25 ng/mL), or anti-CD3/CD28 (1 μg/mL) for 4 days. Tim-3 expressions were assessed on CD4^+^ and CD8^+^ T cells. **(A)** Comparison of frequencies for CD4^+^Tim-3^+^ cells in response to γc cytokine and anti-CD3/CD28 stimulations in AsC. **(B)** Comparison of frequencies for CD4^+^Tim-3^+^ cells in response to γc cytokine and anti-CD3/CD28 stimulations in CHB. **(C)** Comparison of frequencies for CD8^+^Tim-3^+^ cells in response to γc cytokine and anti-CD3/CD28 stimulations in AsC. **(D)** Comparison of frequencies for CD8^+^Tim-3^+^ cells in response to γc cytokine and anti-CD3/CD28 stimulations in CHB. Data were presented as box-and-whisker plot. The box presented as median and quartile, and the whisker plot presented as 2.5–97.5% percentile. Dunn's multiple comparison test were used for comparison between groups.

The common γc cytokines were reported to robustly enhance the Tim-3 expression on CD4^+^ and CD8^+^ T cells in HIV-1 infection (Mujib et al., 2012). Thus, PBMCs from 18 of AsC and 20 of CHB, which were selected from the above experiments, were stimulated for 4 days with various common γc cytokines (including IL-2, IL-7, IL-15, and IL-21), and Tim-3 expression was analyzed on CD4^+^ and CD8^+^ T cells compared with cells in plain medium alone in AsC (CD4^+^, 3.41 ± 2.03%, Figure 2A; CD8^+^, 2.31 ± 1.70%, Figure 2C) and CHB (CD4^+^, 5.77 ± 4.20%, Figure 2B; CD8^+^, 4.57 ± 3.83%, Figure 2D). Tim-3 expression in response to common γc cytokines exhibited similar trends in patients with AsC and CHB. CD4^+^ T cells stimulated with γc cytokine IL-2 (AsC, 6.82 ± 5.85%, *P* = 0.0052; CHB, 10.67 ± 8.71%, *P* = 0.0062; compared with untreated cells), IL-7 (AsC, 4.52 ± 5.37%, *P* = 0.041; CHB, 10.54 ± 8.82%, *P* = 0.011), and IL-21 (AsC, 6.28 ± 5.86%, *P* = 0.033; CHB, 10.58 ± 8.88%, *P* = 0.008) presented remarkable elevated frequencies of Tim-3^+^CD4^+^ T cells (Figures 2A,B). IL-15 stimulation significantly increased the Tim-3^+^CD4^+^ T cells frequency in CHB patients (10.92 ± 7.92%, *P* = 0.008; Figure 2B) but not in AsC patients (5.85 ± 6.31%, *P* = 0.114; Figure 2A). CD8^+^ T cells stimulated with IL-2 (AsC, 4.04 ± 3.48%, *P* = 0.008; CHB, 6.92 ± 5.23%, *P* = 0.0039) and IL-15 (AsC, 4.30 ± 3.36%, *P* = 0.0036; CHB, 9.92 ± 6.43%, *P* = 0.0025) revealed notably increased Tim-3^+^CD8^+^ T cells frequencies (Figures 2C,D). IL-7 stimulation significantly elevated Tim-3 expression on CD8^+^ T cells in CHB patients (6.60 ± 4.62%, *P* = 0.0032; Figure 2D) but not in AsC patients (3.45 ± 3.15%, *P* = 0.250; Figure 2C). However, IL-21 did not increase the frequencies of Tim-3^+^CD8^+^ T cells in either AsC (3.22 ± 3.07%, *P* = 0.053; Figure 2C) and CHB patients (6.33 ± 5.87%, *P* = 0.082; Figure 2D). Furthermore, TCR-stimulated T cells via CD3/CD28 costimulation notably up-regulated Tim-3 expression on both CD4^+^ (AsC, 6.83 ± 5.08%, *P* = 0.001; CHB, 14.46 ± 7.47%, *P* < 0.0001) and CD8^+^ T cells (AsC, 4.52 ± 3.84%, *P* = 0.0015; CHB, 12.30 ± 7.29%, *P* = 0.0015).

### Common γc cytokines did not enhance the Tim-3 induction by either Anti-CD3/CD28 or HBV peptides stimulation

**Figure 3:**
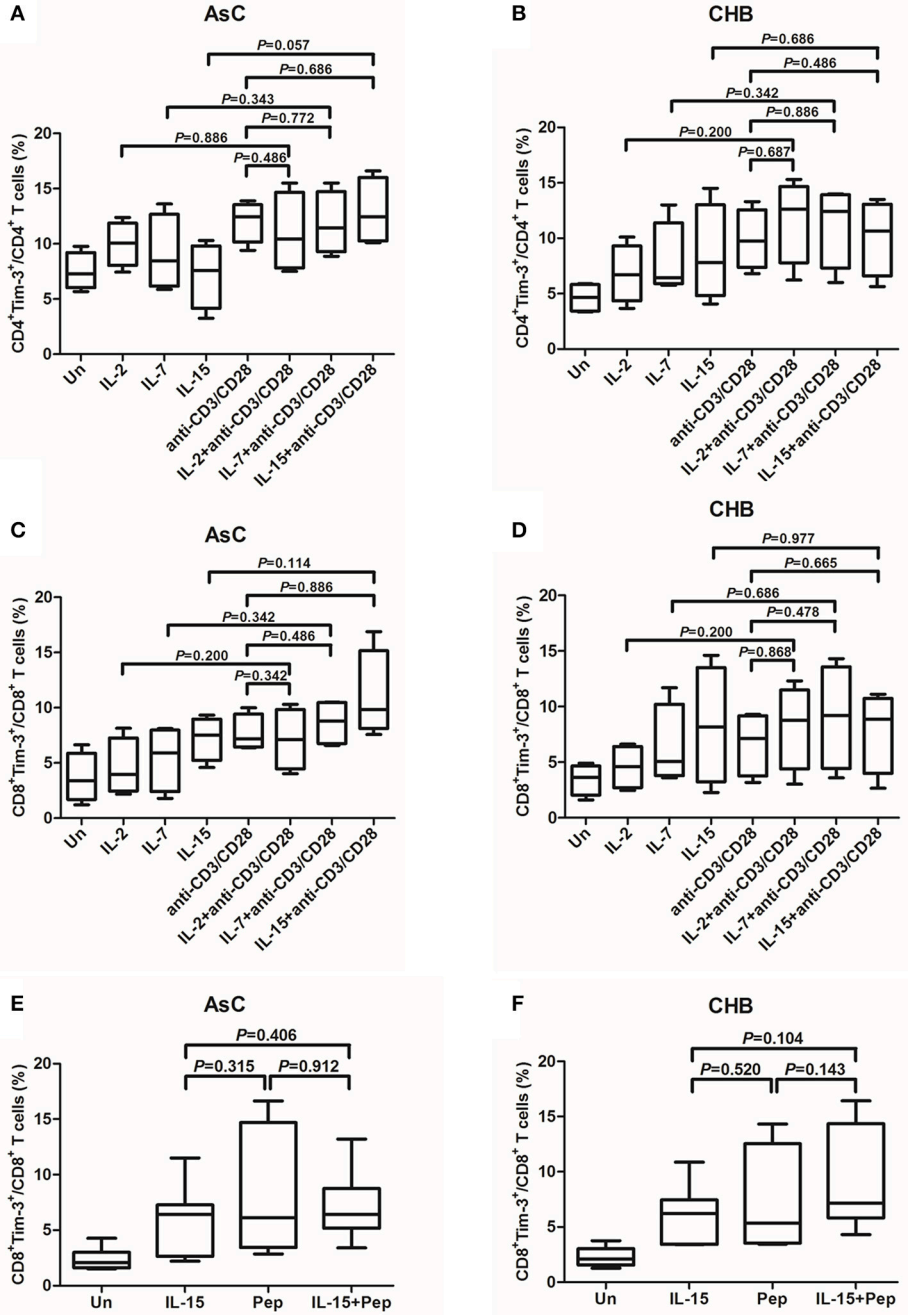
**Induction of Tim-3 expression on T cells within PBMCs in response to common γ-chain (γc) cytokine plus antigens costimulation**. PBMCs were selected from 10 of AsC and 10 of CHB, which were used in the experiments of Figure 1 but did not overlap with the patients from Figure 2. Total PBMCs were treated with IL-2 (25 ng/mL), IL-7 (25 ng/mL), IL-15 (25 ng/mL) plus anti-CD3/CD28 (1 μg/mL) or HBV peptide pools (10 μg/mL). Tim-3 expressions were assessed on CD4^+^ and CD8^+^ T cells. **(A)** Comparison of frequencies for CD4^+^Tim-3^+^ cells in response to stimulation of γc cytokines and costimulation of γc cytokines plus anti-CD3/CD28 in AsC. **(B)** Comparison of frequencies for CD4^+^Tim-3^+^ cells in response to stimulation of γc cytokines and costimulation of γc cytokines plus anti-CD3/CD28 in CHB. **(C)** Comparison of frequencies for CD8^+^Tim-3^+^ cells in response to stimulation of γc cytokines and costimulation of γc cytokines plus anti-CD3/CD28 in AsC. **(D)** Comparison of frequencies for CD8^+^Tim-3^+^ cells in response to stimulation of γc cytokines and costimulation of γc cytokines plus anti-CD3/CD28 in CHB. **(E)** Comparison of frequencies for CD8^+^Tim-3^+^ cells in response to IL-15 stimulation and costimulation of IL-15 plus HBV peptide pools in AsC. **(F)** Comparison of frequencies for CD8^+^Tim-3^+^ cells in response to IL-15 stimulation and costimulation of IL-15 plus HBV peptide pools in CHB. Data were presented as box-and-whisker plot. The box presented as median and quartile, and the whisker plot presented as 2.5–97.5% percentile. Dunn's multiple comparison test were used for comparison between groups.

The γc cytokines IL-2, IL-7, and IL-15 stimulation were more potent inducers of Tim-3 on T cells and, thus, were used for study further. We analyzed whether the γc cytokines presented synergic effect to anti-CD3/CD38 or peptides stimulation on Tim-3 expression on T cells. PBMC from 10 of AsC and 10 of CHB patients, which were selected from the above experiments of Figure 1 but did not overlap with the patients from Figure 2, were cultured with anti-CD3/CD28, alone or presence of either IL-2, IL-7, or IL-15. Tim-3 levels were assessed 4 days after stimulation. The addition of γc cytokines IL-2, IL-7, or IL-15 costimulation with anti-CD3/CD28 did not result in an increased frequencies of either Tim-3^+^CD4^+^ T cells (Figures 3A,C) or Tim-3^+^CD8^+^ T cells (Figures 3B,D). Similar observations were made with IL-15 and cells stimulated with HBV peptides pool. Tim-3^+^CD8^+^ T cells frequencies did not elevated in response to IL-15 and peptides costimulation in either AsC (7.17 ± 3.25%, *P* = 0.912; Figure 3E) or CHB (9.06 ± 4.53%, *P* = 0.143; Figure 3F).

### Common γc cytokines-mediated Tim-3 induction could not be abrogated by γc cytokine receptor-neutralizing antibody

**Figure 4:**
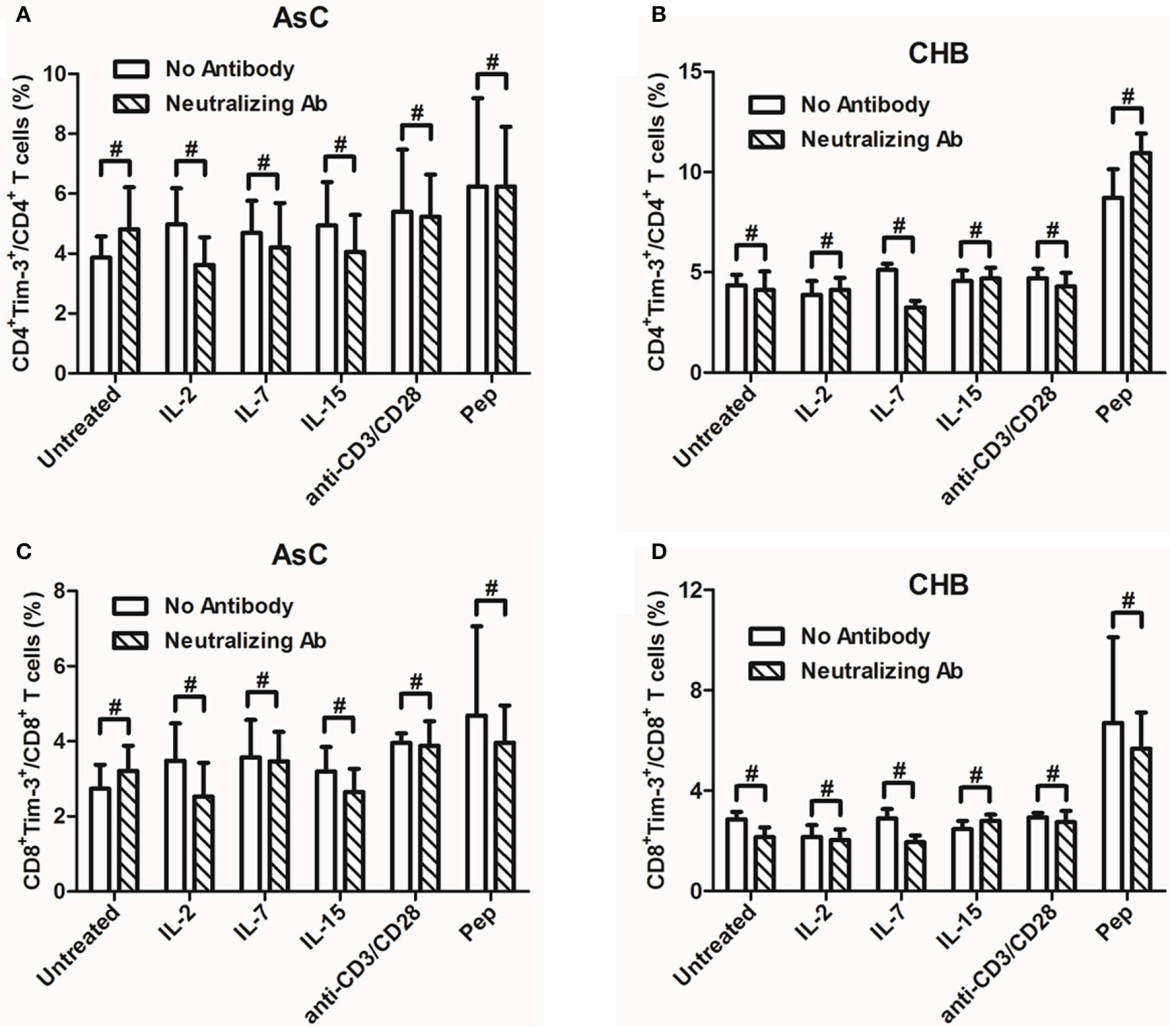
**Induction of Tim-3 expression on T cells within PBMCs by common γ-chain (γc) cytokines (IL-2, IL-7, IL-15) could not be abrogated in the presence of anti-common γc receptor neutralizing antibody compared with no antibody treatments**. PBMCs were selected from 8 of AsC and 8 of CHB patients, which were used in the experiments of Figure 1 but did not overlap with the patients from Figures 2, 3. **(A)** Comparison of frequencies for CD4^+^Tim-3^+^ cells in response to γc cytokines stimulation with or without neutralizing antibody in AsC. **(B)** Comparison of frequencies for CD4^+^Tim-3^+^ cells in response to γc cytokines stimulation with or without neutralizing antibody in CHB. **(C)** Comparison of frequencies for CD8^+^Tim-3^+^ cells in response to γc cytokines stimulation with or without neutralizing antibody in AsC. **(D)** Comparison of frequencies for CD8^+^Tim-3^+^ cells in response to γc cytokines stimulation with or without neutralizing antibody in CHB. Data were presented as mean and standard deviation. Mann-Whitney test was used for comparison between groups. ^#^*P* > 0.05.

The γc cytokines increased Tim-3 expression on T cells through the γ chain of the receptor in HIV-1 infection (Mujib et al., 2012). We then further analyzed whether the signaling through γ chain was also the pathway to regulate Tim-3 expression in HBV infection. PBMCs from 8 of AsC and 8 of CHB patients, which were selected from the above experiments of Figure 1 but did not overlap with the patients from Figure 2 or Figure 3, were cultured with anti-common γc receptor neutralizing antibody at 10 μg/mL for 4 h, and then γc cytokines, anti-CD3/CD8, as well as HBV peptides were added for another 4 days treatment. Compared with the PBMCs which did not receive γc-neutralizing antibody, neither CD4^+^ nor CD8^+^ T cells displayed reduced frequencies of Tim-3^+^ cells with each cytokine stimulation (*P* > 0.05, Figure 4). There were consistent trends of reductions of Tim-3 expression in AsC patients in response to IL-2 (CD4^+^, 4.98 ± 2.40% to 3.62 ± 1.85%, *P* = 0.194, Figure 4A; CD8^+^, 3.49 ± 1.98% to 2.53 ± 1.79%, *P* = 0.199, Figure 4C) and in CHB patients in response to IL-7 (CD4^+^, 2.89 ± 0.74% to 1.94 ± 0.56%, *P* = 0.208, Figure 4B; CD8^+^, 5.13 ± 0.61% to 3.26 ± 0.65%, *P* = 0.051, Figure 4D), but these differences failed to achieve significances. Moreover, both TCR-stimulated cells via anti-CD3/CD28 treatment and viral-specific cells via HBV peptides stimulation were unaffected with regard to Tim-3 frequencies, despite the addition of γc-neutralizing antibody (*P* > 0.05, Figure 4).

**Figure 5:**
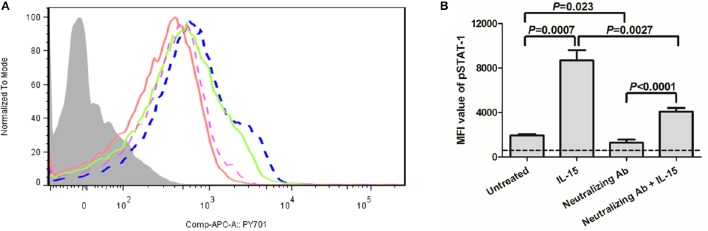
**Induction of phosphorylated STAT-1 in response to IL-15 stimulation in the presence of anti-common γc receptor neutralizing antibody**. PBMCs were selected from 10 of CHB patients which were enrolled in Figure 1. **(A)** Representative histogram of pSTAT-1 were shown. The light gray represented isotype control. The purple dashed line represented untreated PBMCs. The blue dashed line represented IL-15 stimulated normal PBMCs. The red line represented γc receptor neutralizing antibody treated PBMCs. The green line represented IL-15 stimulated, γc receptor neutralizing antibody treated PBMCs. **(B)** Comparison of mean fluorescence intensity (MFI) value of pSTAT-1 among groups. The dashed line represented background levels from isotype control. Data were presented as mean and standard deviation. Mann-Whitney test was used for comparison between groups.

We then further analyzed the phosphoylation of STAT-1 in γc receptor-mediated signaling pathway. PBMCs were selected from 10 of CHB patients which were enrolled in Figure 1. As shown in Figure 5A, IL-15 stimulation significantly increased the mean fluorescence intensity (MFI) value of pSTAT-1(blue dashed line) in comparison with normal PBMCs (purple dashed line) (*P* = 0.0007, Figure 5B). Importantly, inhibition of γc receptor by neutralizing antibody significantly reduced the phosphoylation of STAT-1 (red line) (*P* = 0.023, Figure 5B), which confirmed the successful blockade of γc receptor. Moreover, although MFI value of pSTAT-1 in IL-15 stimulated, γc receptor neutralized PBMCs (green line) was reduced in comparison with IL-15 stimulated normal PBMCs (*P* = 0.0027, Figure 5B), it is still remarkably elevated in comparison of MFI value in γc receptor neutralized PBMCs (*P* < 0.0001, Figure 5B).

## Discussion

In the present study, we provided the evidence to further insight into the mechanism of Tim-3 regulation in chronic HBV infection. We found a higher expression of Tim-3 on both CD4^+^ and CD8^+^ T cells in patients with CHB compared to NCs and AsC patients. This is consistent with the previous notion that Tim-3 revealed a stepwise elevation with increasing liver inflammation which was assessed by ALT levels (Nebbia et al., 2012). Although we did not find significant correlation between Tim-3 expression and HBV DNA or ALT levels in HBV infected individuals, both immunological and inflammatory factors might contribute to the regulation of Tim-3 expression in HBV infection.

HBV-encoding antigens were strong immunogens to activate and stimulate immune cells. Thus, we postulated that soluble HBV viral products could also induce Tim-3 expression. The mixture of HBsAg, HBeAg, and HBcAg were used to stimulate cultured PBMCs *in vitro*. However, Tim-3-expressing CD4^+^ and CD8^+^ T cells did not elevated in response to antigens in both AsC and CHB patients. Furthermore, frequencies of Tim-3 expression were remarkably increased in response to HBV peptide pools stimulation. This is partly because that T cells recognized a peptide derived from the foreign antigen bound to MHC molecule. However, HBV-encoding antigens were unmodified native proteins with different conformations, which did not expose the epitopes recognized by MHC molecules. Moreover, direct stimulation of TCR by anti-CD3/CD28 also notably increase Tim-3 expression on CD4^+^ and CD8^+^ T cells in both AsC and CHB. Thus, Tim-3 expression in HBV-infected individuals was partly an antigen-dependent manner as a result of infection.

Previous studies have been demonstrated that Tim-3 could also be up-regulated both dependently and independently of TCR or antigenic stimulation in viral infection (Hastings et al., 2009; Mujib et al., 2012). Mujib et al. (2012) revealed that Tim-3 could be upregulated *in vitro* in an inflammatory states where enrichment of γc cytokines in HIV-1 infection. Our observation that γc cytokines, specifically IL-2, IL-7, IL-15, and IL-21 were potent inducers of Tim-3 expression on T cells in the antigen-independent manner in HBV infection were consistent with the role of these cytokines in HIV-1 infection (Mujib et al., 2012). The elevations of γc cytokines were proved to be associated with spontaneous viral clearance and HBeAg seroconversion (He et al., 2013). γc cytokines predominantly related to the regulation of lymphocyte development, homeostasis, and functions (Overwijk and Schluns, 2009). IL-2 was a potent inducer of T cell proliferation as well as Th1/Th2 differentiation in inflammatory response (Hoyer et al., 2008). Both IL-7 and IL-15 robustly expanded dendritic cell-activated HBV-specific CD4^+^ T cells *in vitro* (Chen et al., 2006). Moreover, IL-15 was also important in the development and homeostasis of memory CD8^+^ T cells, NK cells, and NKT cells (Villinger et al., 2004). IL-15 also inhibited HBV replication via IFN-β production and exerted anti-HBV functions independent of γc receptor in mouse model (Yin et al., 2012). IL-21, which derived from HBV-specific CD4^+^ T cells, played viral roles in sustaining viral-specific CD8^+^ T cells and promoting B cell response (Li et al., 2015), although our previous studies showed that IL-21 did not enhance HBV-specific immune response in mouse models (Zhang et al., 2014). Importantly, high serum IL-21 levels after 12 weeks of telbivudine therapy predicted HBeAg seroconversion in CHB (Ma et al., 2012). Thus, the up-regulation of Tim-3 in response to γc cytokines(IL-2, IL-7, IL-15, and IL-21) stimulation indicated that Tim-3 may play a negative regulatory role in response to these cytokines, which were consistent with the previously proposed roles of Tim-3 expression on T cells (Sakuishi et al., 2011; Mujib et al., 2012). Although γc cytokines were considered as proinflammation, the involvement of these cytokines in up-regulation of Tim-3 suggested that they were also responsible for activation of inhibitory pathway in viral infections (Mujib et al., 2012). Furthermore, cells costimulated with γc cytokines plus anti-CD3/CD28 or HBV peptides did not result in greater Tim-3 induction compared with mono-stimulation, which suggested that the antigen-dependent and independent induction did not reveal synergic effects in Tim-3 regulation and these two pathways individually were sufficient for Tim-3 induction.

We were not able to diminished γc cytokines-induced Tim-3 elevation on T cells by anti-common γc receptor neutralizing antibody in patients with chronic HBV infection. γc cytokines shared γc receptor usage and signal through specific heterodimeric or trimeric receptor complexes (Toe et al., 2013). The consequences of cognate receptor engagement were dependent on receptor expression patterns, expression levels, and downstream JAK-STAT signaling components (Toe et al., 2013). Thus, downregulation of phosphorylated STAT-1 demonstrated successful blockade of γc receptor. The neutralizing antibody might only partly block the function of common γc receptor. However, other components of the receptor complex might play important roles in γc cytokines-induced Tim-3 expression. This was partly because that IL-15 stimulation could also increase the pSTAT-1 level in γc receptor-inhibited PBMCs. Other possibility could be that γc receptor neutralizing antibody caused a shift in the functional status of Tim-3^+^ T cells. Moreover, γc cytokines might also modulate Tim-3 expression through other signaling pathways. Thus, further studies were needed to investigate the STAT phosphorylation and the changes of Tim-3 expression by functional blocking the other component of receptor complex.

In conclusion, both HBV peptides and γc cytokines induced the up-regulation of Tim-3, which suggested that elevation of Tim-3 expression on T cells could be regulated by both antigen-dependent and independent manner in patients with chronic HBV infection. The role of γc cytokines in modulation of inhibitory pathway could be evaluated as immnotherapies in humans.

## Author contributions

JD, XY, and HS performed the study. XW, LW, C-XH, and YZ enrolled the patients. JD, XW, C-QH, LW, AW, C-XH, YZ, and JL analyzed the data, and prepared the manuscript. YZ and JL designed and supervised the study.

## Funding

This work was supported by the grants from National Natural Science Foundation of China (31370856, 81671555, and 81072353), and National Science and Technology Major Project of China (2012ZX10002007-001-006).

### Conflict of interest statement

The authors declare that the research was conducted in the absence of any commercial or financial relationships that could be construed as a potential conflict of interest.
